# Does Quantity Matter? How Extracurricular Activities Affect Working Memory Development among 5–7-Year-Old Children

**DOI:** 10.11621/pir.2025.0308

**Published:** 2025-09-15

**Authors:** Margarita N. Gavrilova, Polina R. Ivenskaya, Ali K. Tekin, Kristina S. Tarasova

**Affiliations:** a Lomonosov Moscow State University, Russia; b Federal Scientific Center for Psychological and Interdisciplinary Research, Moscow, Russia; c Western Norway University of Applied Sciences, Bergen, Norway

**Keywords:** executive function, working memory, extracurricular activities, 5–7-year-old children, verbal working memory, visual-spatial working memory

## Abstract

**Background:**

It is widely recognized that sports, dance, and other structured extracurricular activities can positively influence children’s executive function. However, previous research has not thoroughly examined whether participation in a diverse range of activities aimed at acquiring new skills affects working memory development in children.

**Objective:**

To investigate the impact of the quantity of extracurricular activities on the development of working memory among 5–7-year-old children.

**Design:**

Longitudinal data on working memory development were collected from children aged 5 to 7 years (N = 101). Three assessments of verbal and visual working memory were conducted at ages 5, 6, and 7 years. Information on children’s participating in extracurricular activities was obtained through parental surveys.

**Results:**

The findings indicate that the number of extracurricular activities in which children participated has a significant positive effect on verbal working memory, with children engaged in multiple types of activities demonstrating a superior ability to retain and reproduce verbal information. Conversely, visual-spatial working memory did not show statistically significant differences based on the number of extracurricular activities.

**Conclusion:**

These results suggest that increasing access to extracurricular activities may foster verbal working memory, which is an important predictor of subsequent academic success and socialization.

## Introduction

Studies in the field of developmental psychology point to the great importance of executive functions in 5–7-year-old children. Executive functions are a group of cognitive abilities that deal with purposeful behavior and problem solving (Veraksa, 2014). There are several theoretical models of executive functions (Cicerone et al., 2006; Miyake et al., 2000; Norman &Shallice, 1986). However, the most accurate model for 5–7-year-old children is the one with three main components: working memory, cognitive flexibility, and inhibitory control (Miyake et al., 2000). These components enable children to successfully focus their attention, remember information of different modalities, develop purposeful activity, planning and other such tasks that are key to a child’s further learning and socialization (Blair, 2002; Welsch et al., 2010; Willoughby et al., 2012).

The first component is working memory, which can be categorized by the type of information perceived: visual working memory and verbal working memory. Visual working memory is involved in retaining and manipulating visual information, while verbal working memory is responsible for processing and storing auditory information and reproducing it. The second component is cognitive flexibility or switching ability. This aspect refers to the ability to quickly and effectively shift attention between different tasks or rules, which is essential for successful adaptation to changing environmental conditions. The third component is inhibitory control. It involves the ability to inhibit habitual or automatic responses in favor of the response required by the task. This is especially important in situations where an alternative response may be incorrect.

Children with well-developed executive functions adapt better to school and perform better academically (Blair & Razza, 2007; Willoughby et al., 2012); manage emotions and behavior more successfully (Denham et al., 2010; Robson et al., 2020); and have higher socio-economic status decades later (Moffitt et al., 2011). Thus, the development of executive functions in the 5–7-year-old children has longterm positive consequences for both the individual and society as a whole. Also there is evidence that targeted training of executive function skills is effective during the preschool years (Schmeichel et al., 2008). However, in order to achieve better results, it is necessary to clarify the scientific understanding of the factors and conditions of their successful formation.

Working memory is one of the key components of executive functions (Miyake et al., 2000). It provides the ability to retain enough information in the child’s mind, which is a necessary condition for the work of other executive functions (Miyake et al., 2000). In studies on a child sample, the development of verbal and visual-spatial working memory is most often assessed (Almazova & Mostinets, 2023; Gavrilova & Chichinina, 2023; Veraksa et al., 2016; Veraksa, 2015). Verbal working memory is the ability to perceive and store verbal information (sounds, words, phrases) in memory. Visual-spatial working memory allows the child to retain new visual information (Baddeley, 1986). Together, these two types of memory are the basis for learning the necessary knowledge and skills.

If a child can retain a sufficient number of items and their relationships to each other in memory, it is easier for the child to solve tasks and learn new material (Halford et al., 1998a). As working memory increases, the speed of information processing, the amount of knowledge, and the strategies available to the child to organize it increase. Thus, weak working memory hinders a child’s ability to be attentive, to focus, and to remember instructions. And vice versa, the more information a child holds in memory, the better he or she navigates a variety of situations and learns (Barkley, 2001; Cowan, 2014; Holmes et al., 2009; Morosanova, 2021; Solovieva, Quintanar, 2015; Verbitskaya et al., 2015).

The above is supported by studies which show that children with working memory deficits often have difficulties with concentration and problem solving (Alloway et al., 2009; Archibald et al., 2011; Aronen et al., 2005; Gathercole et al., 2008; Lui & Tannock, 2007; Pimperton & Nation, 2014). These difficulties impact not only the learning process, but also the child’s daily life. For example, children with poor working memory have difficulty organizing and planning their time and overcoming impulsivity (Gathercole et al., 2008).

One of the components of working memory important to the present study is the phonological loop, which plays a significant role in the process of remembering and repeating information (Baddeley &Hitch, 1974; Baddeley, et al., 1998). The phonological loop is responsible for temporarily storing sound information and repeating it to maintain the integrity of the text read or heard. The phonological loop has two components and is a pholonogical, which is related to speech perception, and an auditory control method, which is related to speech production (Henry, et al., 2012).

Working memory, like other cognitive processes, can be influenced not only by innate abilities, but also by the environment in which a child grows and develops, as well as by his or her activities (Ericsson & Kintsch, 1995). From the perspective of the formation of cognitive abilities, this point can be assessed as promising, since there are reasons to believe that working memory can be developed purposefully by creating conditions that will stimulate its development. However, it should be understood that unfavorable conditions, such as a poor educational environment, can slow the development of working memory (Engel de Abreu et al., 2013; Gavrilova & Chichinina, 2023; Nekipelova &Nekipelov, 2023).

There are several main factors that can affect the development of working memory in children. According to Piaget’s theory of cognitive development, working memory increases as logical thinking develops (Piaget, 2003). It is easier for children to remember new information if they link the new material with what they already know and are able to systematize the knowledge they gain. In general, increasing the amount of knowledge helps to build a more holistic view of a subject and allows the child to better understand the material. According to the principles of the cultural-historical approach, as the child masters cultural knowledge, memory is transformed from a natural mental function into a higher mental function (Vygotsky, 1984). This means that it will be easier for a child to remember new material if he or she has the cultural knowledge necessary to analyze and comprehend new information. This knowledge can include language, writing, and symbols, which work effectively in teaching 5–7-year-old children. Also, according to Activity Theory, a child is able to learn well in meaningful and well-organized activities (Leontiev, 2004). Not only is the structured information presented to the child important, but also his or her personal motivation and involvement in a particular activity. Studies have repeatedly considered the thesis that children demonstrate well-developed executive functions in general and working memory in particular when the quality of lessons and the professionalism of teachers is high and when there is a positive relationship between the teacher and the child. All of these affect the child’s engagement in the learning process and therefore the development of working memory (Bukhalenkova et al, 2022; Egert et al 2018; Howes et al, 2008).

Almost all these factors of successful formation of working memory are present not only in kindergarten or school, but also when a child participates in extracurricular activities. Such activities include musical, sports, creative, language, and theatre activities, which are discussed in more detail in the next subsection.

In addition to the main educational program of a kindergarten or school, extracurricular activities can be useful. For example, dance, sports, language, and other extracurricular activities provide an opportunity for the child to acquire not only new structured knowledge, but also to master certain cultural tools, gain experience of inclusion in different activities and building relationships within different groups (Baddeley et al., 1998; Bayanova et al., 2023; Chichinina et al., 2022; Frischen et al, 2019; Frolli et al., 2022; Gathercole & Baddeley, 1990; Shen et al., 2020; Tvardovskaya et al., 2020).

Musical activities require the child to analyze musical pieces and understand the mood of the music, which requires retention of suffciently large compositional fragments in memory (Bayanova et al., 2023; Frischen et al., 2019; Sepúlveda-Durán et al., 2024; Veraksa et al., 2023). Dance activities engage working memory because the child needs to learn the sequence of movements and relate them to rhythm and melody (Chichinina et al., 2022; Shen et al., 2020). Learning foreign languages promotes the development of working memory not only through learning new words, but also through the formation of new concepts that arise from generalizing concepts already known to the child (Baddeley et al., 1998; Gathercole & Baddeley, 1990). More to the point, sports activities are also beneficial for child development, as the child needs to memorize the correct execution of compound cardiorespiratory movements, act according to the instructions received, and memorize the coach’s instructions (Frolli et al., 2022; Tvardovskaya et al., 2020; Veraksa et al., 2021; Veraksa et al., 2020; Zarazaga-Peláez et al., 2024). Thus, extracurricular activities, whether chess, music, sports or dance, imply mastering not only new knowledge but also specific complex skills.

In addition to the content that children learn in extracurricular activities, it can also be beneficial for working memory if these activities are often organized in a group format. Group work involves interaction between children, with discussion and sharing of ideas, which helps to stimulate mental activity. Group learning encourages repetition and consolidation of information learned, which is also beneficial to the development of working memory (Diamond &Ling, 2020).

It is also important that participation in extracurricular activities structures the child’s daily life. Systematic attendance and preparation for activities stimulates the child’s working memory training. When a child follows a daily routine, the need to retain the tasks in memory helps the child to successfully carry out his or her activities. Additional activities become even more valuable if they ensure that the child is emotionally involved and enjoys the learning process (Vinogradova, 2004). This strengthens motivation and the desire to learn. Positive emotions associated with learning can significantly increase the effciency of memorizing new material.

Thus, well-structured and organized extracurricular activities allow children not only to successfully learn new material but also to spend time in a pleasant and useful way, particularly in terms of working memory training. This, in turn, lays a solid foundation for their further learning and development.

This longitudinal study examines changes in working memory associated with children’s participating in extracurricular activities (beyond the compulsory kindergarten or school program), while controlling for confounding factors such as gender, age, and some other socio-demographic variables. A longitudinal perspective analyzed the development of working memory in children aged 5 to 7 years with three measurement points conducted in 2022 (Time 1), 2023 (Time 2), and 2024 (Time 3). Data were collected on measures of verbal and visual working memory; on the number of extracurricular activities in which the children participated; and the number of children in the family, the mother’s education level, and the family income at the time of the study. The main hypothesis of the study was that the more extracurricular activities a child participates in in addition to the main kindergarten or school program, the better the development of his/her verbal and visual-spatial working memory, both at the age of 5 and at the age of 7.

## Methods

### Participants

The participants were 5–7-year-old children from Moscow from 2022 to 2024. The initial number of children included in the study was 135. However, after applying the inclusion criteria (full-term birth, birth weight over 2,500g, absence of visual or hearing impairment, and availability of data from the parental questionnaire and all three assessment waves). To be included in the final sample, a child had to have complete data from all three assessments (at ages 5, 6, and 7); children with missing data for any of the time points were excluded). As a result of applying these criteria, 34 children were excluded from the analysis. The final sample included 101 children. At the time of the first assessment, the average age of the children was 5 years (M = 63.6 months, SD = 3.29). A second assessment involving the same children was conducted one calendar year later, at the age of 6 (M = 75.4 months, SD = 3.72). The third assessment was conducted at the age of 7 (M = 90.3 months, SD = 6.12).

Information about participating in extracurricular activities beyond the compulsory kindergarten program was obtained from a survey of parents. Only 14% of the children participating in the study did not participate in any extracurricular activities (of which 42.9% were boys and 57.1% were girls). The remaining 87 children (86% of the total sample) participated in extracurricular activities: 33 children participated in 1 extracurricular activity (33% of the total sample), 21 children participated in 2 extracurricular activities (21% of the total sample), 21 children participated in 3 extracurricular activities (21% of the total sample), and 12 children participated in 4 or more extracurricular activities (12% of the total sample). Of these, 63.4 % of children participated in sports activities; school preparation activities, 46.5%; fine arts and arts and crafts, 3.7%; dance, 33.7%; and chess, 11.9%. The sample included only those children who participated in extracurricular activities consistently throughout all three years of the study.

As a result of the survey of parents about socio-demographic characteristics of families, the following data were obtained. The majority of respondents classified their family as having an average income (74.3%); 91.1% of mothers had higher professional education. At the time of the survey, 27.7% of the participants were the only children in the family; 5.5% grew up with one brother or sister, and the remaining 21.8% were raised with two or more siblings.

### Measures

The *Sentences Repetition subtest* of the NEPSY-II was used to assess verbal memory (Korkman et al., 2007). This instrument is a list of 17 sentences that gradually become more difficult to remember (as the sentences increase in length and become more grammatically complex). The maximum score on the instrument is 34.

The *Memory for Designs subtest* of the NEPSY-II was used to assess visual-spatial working memory (Korkman et al., 2007). The test includes four tasks of varying complexity, in which the child must remember the picture shown for 10 seconds and then select and place multicolored cards with images on a special frame (4x4 cells) in the same configuration in which they were arranged on the picture initially presented. The selection set of cards includes distractors. The maximum score is 12.

A parent questionnaire was designed and administered to collect data on the number of extracurricular activities in which the children participated beyond the compulsory kindergarten program, inclusion criteria for the sample (full-term birth, birth weight, absence of visual or hearing impairment), and socio-demographic characteristics of families (family income, mother’s education, and presence of siblings).

### Procedure

Assessment was conducted in kindergartens and schools, in a calm environment that excluded distraction of the participants’ attention. Assessment was carried out individually. All testers were trained before the survey and followed the standard principles of psychological assessment of 5–7-year-old children. Assessment was carried out three times at intervals of one year. At the time of the first assessment, the average age of the children was 5 years. The second assessment involving the same children was conducted one calendar year later, at the age of 6. Then the third assessment was conducted at the age of 7.

## Results

Descriptive statistics for the results of assessment of verbal working memory and visual working memory of the children are presented in *Table 1*. The results are presented separately for each age at which the examination was conducted (5, 6 and 7 years) and are divided by the number of extracurricular activities in which the children participated.

**Table 1:** Descriptive Statistics for the Main Variables Examined in the Study

Variable	5–6 y.o.				6–7 y.o.				7–8 y.o.			
	M	SD	Min	Max	M	SD	Min	Max	M	SD	Min	Max
Verbal working memory (without extracurricular activities)	16.3	4.29	8	25	18.2	3.03	15	25	21.6	3.61	14	29
Verbal working memory (with 1 extracurricular activity)	16.5	4.41	0	26	18.9	3.28	12	27	21.2	2.96	15	28
Verbal working memory (with 2 extracurricular activities)	17.5	4.12	9	24	20	4.27	13	26	21.8	3.8	14	28
Verbal working memory (with 3 extracurricular activities)	18.4	3.05	15	26	20.8	4.22	14	29	23.4	3.8	16	31
Verbal working memory (with 4 or more extracurricular activities)	19.2	3.71	14	26	21.5	3.42	16	27	24.3	4.56	16	31
Visual working memory (without extracurricular activities)	73.2	17.37	48	101	88	15.65	61	113	113.1	26.74	36	144
Visual working memory (with 1 extracurricular activity)	68.3	20.29	38	115	87.8	23.21	44	120	114.2	26.06	41	151
Visual working memory (with 2 extracurricular activities)	67.1	18.1	40	107	90.3	19.62	60	120	105.7	34.68	39	148
Visual working memory (with 3 extracurricular activities)	70.7	19.21	34	106	80.9	14.13	51	114	106.3	33.38	35	148
Visual working memory (with 4 or more extra- curricular activities)	73.3	19.72	32	99	92	16.74	69	117	107.9	28.97	34	136
Age	63.6	3.29	54	73	74.4	3.72	68	84	90.3	6.12	78	102

**Table 2:** Results of the Analysis of Within-Subject Effects (*Changes over Three Years of Measurement*) for Dependent Variables

Variable	Age 5–7		
	F	p	η^2^
Verbal working memory	97.113	<.001	.199
Visual working memory	89.243	<.001	.347

**Figure 1.**
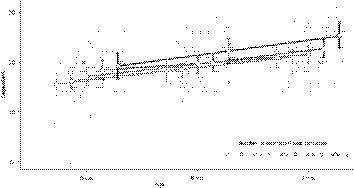
Development of verbal working memory from 5 to 7 years, depending on the number of activities in which children participated in addition to the compulsory kindergarten or school educational program.

The main hypothesis of the study, that the capacity of verbal and visual-spatial working memory of children aged 5 and 7 depends on the number of extracurricular activities in which they participated, was tested by means of a comparative analysis of variance with repeated measures (Repeated Measures ANOVA). The dependent variables were the results of the verbal and visual-spatial working memory assessment from the first, second, and third assessments, and the independent variable was the number of extracurricular activities in which the child participated. The analysis revealed that the number of extracurricular activities had a significant positive effect on the ability to hold information in verbal working memory (F(4) = 2.73, *p* = .035, η^2^= .082) (see *Figure 1*). However, visual-spatial working memory was not statistically significantly different according to the number of extracurricular activities in which children participated (F(4) = .290, *p* = .884, η^2^= .006).

In order to controlling for potential confounding variables, the role of sociodemographic factors in the development of working memory and the number of classes in which children participated were also analyzed. The presence of siblings, according to the results obtained by comparative analysis of variance with repeated measures (Repeated Measures ANOVA), statistically significantly conditioned verbal (F(2) = 4.72, *p* = .012, η^2^= .045), but not visual-spatial (F(2) = 1.53, *p* = .222, η^2^= .014) working memory development. Family income and mother’s education level did not directly influence working memory development (*p* > .05). The role of socio-demographic factors in the number of extracurricular activities in which the children participated was analyzed using Pearson’s X^2^. A statistically significant relationship was found between the number of extracurricular activities children participated in and the mother’s educational level (X^2^ = 3.7; *p* = .015), family income (X^2^ = 17.5; *p* = .025), and the presence of siblings (X^2^ = 15.4; *p* = .052).

## Discussion

In this study, aimed at examining an important factor in the development of children’s cognitive abilities in 5–7-year-old children, we asked about the benefits of children participating in extracurricular activities for working memory development. The benefits of extracurricular activities for working memory development have been pointed out by many authors, including evidence that learning complex activities, such as playing a musical instrument or other specific skills, affects brain anatomy (Baker et al., 2003; Cross et al., 2006; Hutchinson et al., 2003; Nielsen &Cohen, 2008; Schlaug, 2001). For example, regular music practice has been shown to increase grey matter volume in motor areas of the brain (Hyde et al., 2009). Such structural differences appear about a year after the beginning of training (Hyde et al., 2009; Schlaug et al., 2005). Retrospective studies have also supported the benefit of extracurricular activities during the childhood years for the development of cognitive executive function skills (Forgeard et al., 2008; Ruthsatz et al., 2008; Schellenberg, 2006). However, such studies have not analyzed the effect of the number of extracurricular activities in which a child participated.

The purpose of this study was to analyze changes in children’s working memory over a three-year period in the context of the number of extracurricular activities in which they participated. It was hypothesized that the more extracurricular activities in which a child participated, the more developed their verbal and visual-spatial working memory was at both age 5 and age 7. In addition, the possible influence of such factors as the presence of siblings, mother’s educational level, and family income on the number of extracurricular activities and working memory was considered.

The study confirmed the hypothesis that verbal working memory scores improved significantly with the increase in the number of extracurricular activities in which a child participated. Children who participated in more than one type of extracurricular activity performed better on the task of memorizing and reproducing verbal information. This pattern is consistent with previous findings on the benefits of extracurricular activities for working memory during the childhood years (Bayanova et al., 2023; Chichinina et al., 2022; Frischen et al., 2019; Frolli et al., 2022; Shen et al., 2020; Tvardovskaya et al., 2020). However, the development of children’s visual-spatial working memory over the entire study period was not influenced by the number of extracurricular activities in which the children participated. This result may indicate different mechanisms underlying the retention of verbal and visual information.

Indeed, according to previously published data, verbal information is stored in memory due to the coordinated work of three brain structures that form the so-called phonological loop (Bergman et al., 2014). The phonological loop ensures that verbal information is processed and retained for short periods of time (Baddeley et al., 1998). It is likely that the development of visual-spatial working memory occurs through more complex psychophysiological mechanisms and takes longer for meaningful changes to emerge.

The results of the current study confirm that working memory may be conditioned by the social environment. Consequently, unfavorable conditions or a poor educational environment may slow the development of working memory, which has also been demonstrated in earlier studies (Nekipelova &Nekipelov, 2023; Gavrilova & Chichinina, 2023; Engel de Abreu et al, 2013).

We also examined how socio-demographic variables and family environment affect the development of working memory in children. Thus, as part of the additional hypothesis testing, the role of such socio-demographic factors as mother’s educational level, family income, and the presence of siblings was examined. It was shown that the mothers of those children who participated in a greater number of extracurricular activities had a higher level of education. Despite the fact that it is objectively more difficult for parents in large families to organize children’s participating in extracurricular activities (including due to inconsistency of school shifts and time of activities), no differences in their number were revealed. At the same time, according to the results obtained, the presence of siblings has a statistically significant positive effect on the development of verbal working memory. This result is also consistent with earlier studies showing that working memory, like other cognitive processes, is determined not only by innate data, but also by the environment in which a child grows and develops (Ericsson & Kintsch, 1995).

However, a child’s life does not have to be all extracurricular activity. Play, including with siblings, is an effective means of developing memory and other intellectual abilities (Greenough &Black, 1992; Han et al., 2022). It has been demonstrated that children, when participating in play activities, activate their cognitive abilities (Bukhalenkova et al., 2021; Veraksa et al., 2020). This is because play is the leading activity in 5–6-year-old children and largely influences the child’s mental development (Vygotsky, 2004). Scientific works indicate that play activities have a positive effect on short-term memory performance and the ability to switch attention (Yogman et al., 2018).

The findings highlight the need to develop recommendations aimed at improving working memory through enriching the child’s educational experience, including participating in extracurricular activities. The development and accessibility of extracurricular education programs for children should be supported. Enriching a child’s experience should have a favorable impact on their ability to remember and retain new auditory, visual, and spatial information (Frolli et al., 2022; Shoghi & Ghonsooly, 2017; Weiland et al., 2013). Also, the results of the study may indicate the need for more detailed study of the factors and mechanisms of visual-spatial working memory development.

## Conclusion

This article presents the results of a longitudinal study of working memory changes associated with children participating in extracurricular activities in addition to the compulsory kindergarten program and school. The results confirmed the assumption that the more activities in which a child participates in addition to the main kindergarten and school program, the better is the development of his/her verbal working memory, both at the age of 5 and the age of 7. However, this pattern was not found for visual-spatial working memory. Since executive functions are one of the predictors of further successful learning and socialization of a child, the results may indicate the need to increase the availability of extracurricular activities for children to create conditions that promote the development of executive functions.

## Limitations

One of the most obvious limitations of this study is the analysis of only the number of extracurricular activities, not their types. Although data were available on what types of activities participants participated in, the decision was made to analyze only the number of activities because of concerns about the statistical power of the results. Thus, attempting to differentially analyze the effect of different types of activities would have resulted in a loss of statistical power, as many children participate in several types of extracurricular activities at once. Future analyses of differential effects will be possible only with a larger study sample. It should also be considered that children attending preschool have developed working memory due to the fact that kindergartens provide activities and games that can positively influence the development of working memory. Another less obvious, but extremely important limitation in the context of the results obtained, is the potential differences in individual psychological characteristics of children. The number of activities in which a child participates depends not only on the parents’ decision and the chosen educational strategy, but also on the child’s temperament. Children with difficult temperaments, such as high negative affect or slowness, are less likely to be motivated to regularly participate in extracurricular activities and more likely to encounter difficulties that reduce their developmental effect.
